# Evaluation of the Glymphatic System Using the DTI-ALPS Index in Patients with Spontaneous Intracerebral Haemorrhage

**DOI:** 10.1155/2022/2694316

**Published:** 2022-07-05

**Authors:** Chao Zhang, Jingyun Sha, Lulu Cai, Yingying Xia, Danyang Li, Houliang Zhao, Chong Meng, Kai Xu

**Affiliations:** ^1^Department of Radiology, Affiliated Hospital of Xuzhou Medical University, Xuzhou, 221000 Jiangsu, China; ^2^School of Medical Imaging, Xuzhou Medical University, Xuzhou, 221000 Jiangsu, China; ^3^Department of Stomatology, The Fifth Affiliated Hospital of Xinjiang Medical University, Xinjiang 830000, China

## Abstract

**Objective:**

To investigate the function of the human glymphatic system (GS) in patients with spontaneous intracerebral haemorrhage (sICH) using diffusion tensor imaging analysis along with the perivascular space (DTI-ALPS).

**Methods:**

Twenty patients with sICH and 31 healthy controls (HCs) were recruited for DTI and susceptibility-weighted imaging scanning. The diffusivity along the perivascular spaces, as well as the projection fibres and association fibres, was evaluated separately. The DTI-ALPS index of each subject was also calculated. Two-sample *t*-tests and paired *t*-tests were performed to analyse the difference in ALPS scores between patients and HCs, as well as that between the lesion side and contralateral side. Pearson correlation analysis was used to observe the relationship between disease duration and GS function.

**Results:**

The DTI-ALPS index on the lesion side was significantly lower than that of the contralateral side in patients with sICH (*p* < 0.01, *t* = −5.77), and it was also significantly lower than that of the ipsilateral side of HCs (*p* < 0.01, *t* = −9.50). No significant differences were found in the DTI-ALPS index on the nonlesion side between patients and HCs (*p* = 0.96, *t* = 0.05) or between the left and right cerebral hemispheres of HCs (*p* = 0.41, *t* = −0.83). The DTI-ALPS index of the lesion side in patients with sICH was significantly correlated with disease duration (*p* = 0.018, *r* = 0.537).

**Conclusions:**

The present study confirmed that GS dysfunction on the ipsilateral side of the lesion is impaired in patients with haemorrhagic stroke, indicating that the GS may be a separate system in the left and right cerebral hemispheres. The DTI-ALPS index can reflect disease duration. These findings have significant implications for understanding sICH from a new perspective.

## 1. Introduction

Spontaneous intracerebral haemorrhage (sICH) refers to a nontraumatic intraparenchymal haemorrhage caused by spontaneous rupture of cerebral blood vessels [1]. Compared with ischaemic stroke, sICH has the characteristics of faster disease progression, higher mortality, and long-term disability, resulting in a heavy social and economic burden [2]. Despite some advances in early prevention and acute interventions over the past decade, the high mortality rate of ICH has not been reduced [3]. Furthermore, sICH survivors still have a high risk of recurrent haemorrhagic and ischaemic stroke [4]. To date, there is still a lack of effective interventions and therapeutic drugs for sICH administration [5]. Therefore, understanding the pathophysiology of sICH and obtaining sICH-related imaging markers may contribute to the exploration of therapeutic approaches and assessments of treatment effects.

The glymphatic system (GS) was first discovered by Iliff et al. [6] through two-photon imaging of small fluorescent tracers in 2012. The GS consists of arteries, aquaporin 4 (AQP4) located on the astrocyte endfeet, and the perivascular space (PVS) [6]. It is a system that promotes the exchange and flow of cerebrospinal fluid (CSF) and interstitial fluid (ISF) mediated by AQP4 in astrocyte endfeet [6, 7]. CSF enters the brain parenchyma through the PVS close to cerebral cortical arteries and perforating arterioles, completes the exchange of substances between CSF and ISF, removes metabolic waste through the PVS [8], and finally moves waste to deep cervical lymph nodes and the peripheral lymphatic system [9]. GS can remove soluble amyloid beta (A*β*) [6], tau protein [10], lipids [11], proinflammatory cytokines, and neurotoxic solutes [12]. The PVS plays a critical role in the entire transport process.

GS is involved in the pathological process of stroke, including brain oedema, blood–brain barrier (BBB) disruption, immune cell infiltration, neuroinflammation, and neuronal apoptosis [13]. Some studies have demonstrated that enlarged PVS (EPVS) is an important factor in the pathogenesis of ICH [14]. As a key anatomic structure of the GS, EPVS was suggested to be closely related to impaired GS clearance of inflammatory substances and metabolic waste in traumatic brain injury [15]. The GS was also reported to be strongly linked with the pathophysiology of other nontraumatic diseases, such as Alzheimer's disease [16], Parkinson's disease [17], and type 2 diabetes mellitus [18], by using diffusion tensor imaging analysis along with the PVS (DTI-ALPS). As suggested by the above studies, DTI-ALPS can be used as a quantitative imaging biomarker to monitor the ability of the GS to remove metabolic wastes, providing a new method for noninvasively evaluating human GS function in vivo [19]. However, fewer studies have focused on the association of the GS with sICH by using the DTI-ALPS index. Thus, the aim of this study was to investigate the pathological features of GS in patients with sICH using this new method of DTI analysis.

## 2. Materials and Methods

### 2.1. Subjects

We recruited a convenience sample of 23 hospitalized patients with sICH. All patients received conservative treatment, and magnetic resonance imaging (MRI) was performed on each patient one month later. The inclusion criteria were as follows: (i) haemorrhage lesion located in the left cerebral hemisphere, and it is at least three slices away from the slice of measured ROI, (ii) no severe cerebral white matter lesions, (iii) no history of head trauma, and (iv) no comatose state a week before and after MRI administration. Patients with one of the following conditions were excluded: (i) MRI revealing lesions in the brain parenchyma other than haemorrhage, (ii) the haemorrhage is too large that it would affect DTI metrics measurement, (iii) cognitive impairment, and (iv) history of neuropsychiatric disease. Thirty-three healthy volunteers were selected as healthy controls (HCs). Inclusion criteria for HCs were as follows: (i) age and sex matched with participants in the patient group, (ii) no symptoms and signs of any neurological disease, and (iii) no history of drug use, alcohol abuse, or mental illness. The exclusion criteria were as follows: (i) intracerebral lesions detected by routine MRI scans and (ii) neurological diseases or a family history of genetic diseases.

This study was performed in accordance with the tenets of the Helsinki Declaration and was approved by the local Ethics Committee of Xuzhou Affiliated Hospital, Xuzhou Medical University. Written informed consent was obtained from all subjects before participation in the study.

### 2.2. MRI Data Acquisition

All subjects underwent 3.0 Tesla MRI scanning (GE Medical Systems, Signa HD, Waukesha, WI, USA) with an eight-channel head coil. Each subject was stabilized with comfortable foam pads to minimize head movement and wore earplugs to prevent the influence of noise during scanning. High-resolution T1-weighted images (T1WIs) were acquired by using a three-dimensional- (3D-) T1 brain volume (BRAVO) sequence, which provided isotropic voxels of 1 mm × 1 mm × 1 mm; repetition time (TR), 7 ms; echo time (TE), 3 ms; field of view (FOV), 256 mm × 256 mm; number of slices, 192; and flip angle, 12°. DTI data were obtained using the following parameters: TR/TE = 9000/90 ms, matrix = 128 × 128 mm^2^, FOV = 256 × 256 mm^2^, number of diffusion gradient directions = 64, *b* value = 1000, and providing voxels of 3 mm × 2 mm × 2 mm. The enhanced gradient echo T2∗ weight angiography (ESWAN) sequence was used for the acquisition of susceptibility-weighted imaging (SWI) data, with the following parameters: TR/TE = 44.5 ms/5.5 ms, bandwidth = ±41.67 kHz, slice thickness/slice spacing was 2 mm/0 mm, and the reversal angle was 15°. In addition, all patients underwent T2WI (TR/TE = 4968 ms/172.2 ms), DWI (TR/TE = 4880 ms/77.3 ms), and FLAIR (TR/TE = 9000 ms/94 ms) scans, with the same FOV = 240 × 240 mm^2^, slice thickness/slice spacing = 6.0 mm/1.5 mm, and the same number of slices = 18.

### 2.3. MRI Data Processing

**Figure 1:**
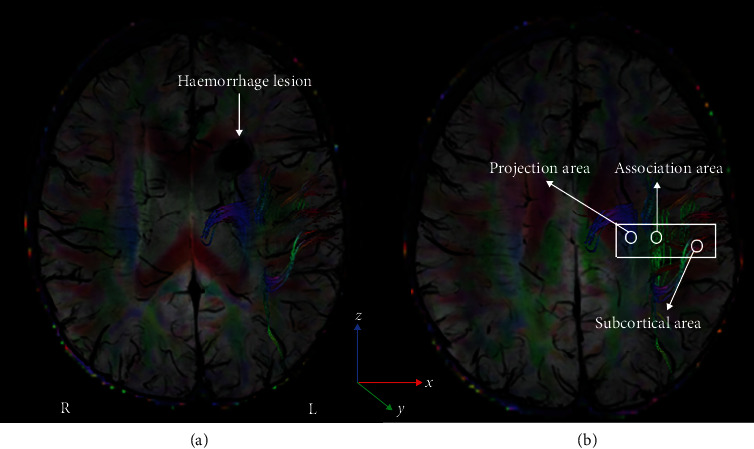
The DTI and SWI fusion colour map shows the directions of the projection fibres (blue; *z*-axis), association fibres (green; *y*-axis), and subcortical fibres (red; *x*-axis). (a) The haemorrhage lesion located near the left lateral ventricle. (b) The ROIs placed to measure DTI parameters of the projection and association fibres. DTI: diffusion tensor imaging; SWI: susceptibility-weighted imaging.

The DTI Studio software was used in this study to measure DTI metrics (https://www.mristudio.org/). Briefly, the steps were as follows: (i) raw DTI data of a single individual were imported into the software; (ii) automatic image registration was performed; and (iii) the diffusion tensor was calculated, including a colour-coded fractional anisotropy (FA) map and diffusivity in the directions of the *x*-axis, *y*-axis, and *z*-axis. The direction of the PVS is perpendicular to the ventricle wall. This direction is also perpendicular to the direction of the projection fibres as well as the association fibres (Figure 1). According to previous studies, we evaluated the diffusivity along the direction of the PVS to further calculate the DTI-ALPS index [17–19]. Given that the PVS runs concentrically along the medullary vein structures at the level of the lateral ventricles, SWI scans were performed to accurately visualize these fine veins. The DTI-FA map was overlaid on SWI scans to precisely draw regions of interest (ROIs) (Figure 1). A 4 mm diameter ROI was placed in the area of the projection fibres and the area of the association fibres in the bilateral hemisphere. For each participant, diffusivity in the directions of the *x*-axis, *y*-axis, and *z*-axis of each area was measured. Two radiologists with 2 years of work experience processed the data of all subjects. Then, each DTI parameter, measured by two radiologists, was averaged as the final result of each individual.

Subsequently, the DTI-ALPS index was calculated by using the DTI parameters measured above. According to a study by Taoka et al. [19], this index is determined by the ratio of two diffusivity value sets, i.e., the ratio of the average values of the *x*-axis diffusivity in the area of the projection fibres (Dxxproj) and the *x*-axis diffusivity in the area of the association fibres (Dxxassoc) to the average value of the *y*-axis diffusivity in the area of the projection fibres (Dyyproj) and the *z*-axis diffusivity (Dzzassoc) of the association fibres area. More specifically, this relationship is defined as follows: DTI-ALPS index = mean (Dxxproj, Dxxassoc)/mean (Dyyproj, Dzzassoc). A lower ratio represents less water diffusivity along the PVS, indicating reduced GS activity.

The haemorrhagic lesions were delineated manually by two radiologists on individual high-resolution T1WI images using ITK-SNAP (http://www.itksnap.org), and the volume of each haemorrhage was automatically generated. Each individual volume obtained from each radiologist was averaged as the final result.

### 2.4. Statistical Analysis

A chi-square test was used to observe sex differences between participants in the sICH and HC groups. Intraclass correlation coefficient (ICC) statistics were used to assess interobserver agreement for DTI parameter measurements. A two-sample *t*-test was employed to find intergroup differences in age and DTI-ALPS index. Differences in the DTI-ALPS index between the left and right cerebral hemispheres of all the subjects were tested using paired *t*-tests. Pearson correlation analyses were carried out to test whether DTI-ALPS data of patients with sICH can indicate disease duration. The threshold for the significance level was set at 0.05.

## 3. Results

### 3.1. Demographics of the Participants

Three patients were excluded because the haemorrhage area was too large and would affect the measurement of DTI parameters, and 2 healthy volunteers were excluded due to obvious head movement. Finally, 20 patients with sICH (13 males and 7 females, 47.8 ± 12.4 years old) and 31 HCs (15 males and 16 females, 47.3 ± 10.9 years old) were included in the present study. No significant difference was found in age (*p* = 0.87) or sex (*p* = 0.24) between participants in the sICH and HC groups. The disease duration was 21-155 (55.6 ± 38.8) days. The haemorrhage volume was 7.4-33.2 (23.92 ± 6.67) mm^3^ (Table 1).

### 3.2. Group Analysis of DTI-ALPS

**Figure 2:**
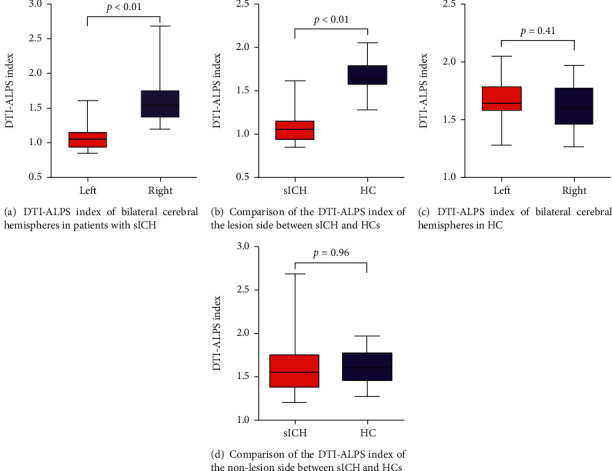
Differences in the DTI-ALPS index between the left and right cerebral hemispheres of patients with sICH and HCs were tested using paired *t*-tests (a, c). Intergroup differences in the DTI-ALPS index were revealed by a two-sample *t*-test (b, d). DTI-ALPS: diffusion tensor imaging analysis along with the perivascular space; sICH: spontaneous intracerebral haemorrhage; HCs: healthy controls.

**Figure 3:**
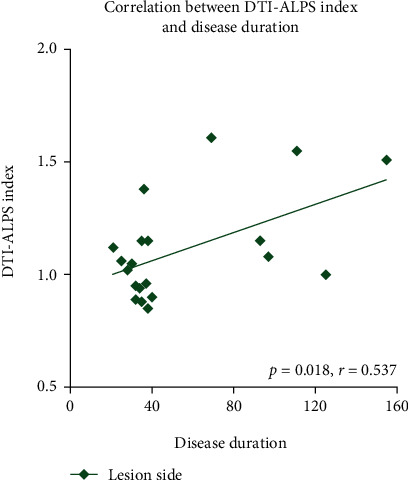
The DTI-ALPS index of the lesion side in patients with sICH was significantly correlated with disease duration (*p* = 0.016, *r* = 0.531). DTI-ALPS: diffusion tensor imaging analysis along with the perivascular space; sICH: spontaneous intracerebral haemorrhage.

High interobserver agreement (ICC = 0.76, *p* < 0.001) was found for the DTI-ALPS calculation of all patients. The DTI-ALPS index of the patient's lesion side (left side) was 1.11 ± 0.23, and the DTI-ALPS index of the contralateral side (right side) was 1.64 ± 0.41. The DTI-ALPS index of the left and right cerebral hemispheres of the HCs was 1.67 ± 0.19 and 1.63 ± 0.19, respectively. The DTI-ALPS index on the lesion side was significantly lower than that of the contralateral side (*p* < 0.01, *t* = −5.77); in addition, DTI-ALPS index on the lesion side was significantly lower than that of the ipsilateral side of the HCs (*p* < 0.01, *t* = −9.50) (Figure 2). No significant differences were found in the DTI-ALPS index on the nonlesion side between patients and HCs (*p* = 0.96, *t* = 0.05) or between the left and right cerebral hemispheres of HCs (*p* = 0.41, *t* = −0.83) (Figure 2). For correlation analysis, we found that the DTI-ALPS index of the lesion side was significantly correlated with disease duration (*p* = 0.018, *r* = 0.537) (Figure 3).

## 4. Discussion

The main goal of the current study was to determine the pathological changes in the GS in patients with sICH by means of DTI analysis. The most obvious findings to emerge from this study were that (i) impaired GS function was revealed on the lesion side in patients with sICH, (ii) the DTI-ALPS index can reflect the disease duration, and (iii) no obvious change was found in the DTI-ALPS index on the nonlesion side in patients with sICH. The current data highlight the importance of the GS in understanding the pathophysiological mechanisms of sICH from a new perspective.

**Figure 4:**
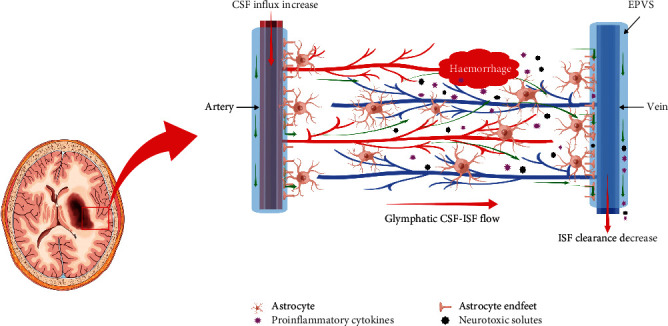
The anatomy and function model of the GS in pathophysiology conditions of sICH. The PVS is enlarged after intracerebral haemorrhage. Abundant proinflammatory cytokines and neurotoxic solutes accumulate in the brain parenchyma secondary to haemorrhage, and the clearance rate of GS is decreased. GS: glymphatic system; sICH: spontaneous intracerebral haemorrhage; EPVS: enlarged perivascular space.

The loose fibrous matrix of the PVS provides a low resistance pathway for CSF-ISF flow and plays an important role in brain fluid transport. Physiological factors, such as arterial pulsation, respiration, and CSF pressure gradient, facilitate CSF entry into the subarachnoid space, allowing it to enter the deep structure of the brain along the PVS. CSF is then mediated by AQP4 into the brain tissue space and promotes the flow of ISF back to the subarachnoid CSF through the PVS and to the surrounding veins within the overall flow. Finally, the metabolites and toxic proteins of CSF flow to the cervical lymph nodes and peripheral lymphatic system through the meningeal lymphatic vessels, which play a key role in maintaining the balance of CSF-ISF and removing metabolic wastes in the brain [20]. The PVS and AQP4 polarity distribution are the two most important structures in this process of circulation and metabolism. Of note, the PVS is a small tissue gap around the cerebral artery and vein. The inner wall is composed of the vascular wall and the outer wall, which consists of the basement membrane (extracellular matrix component) (Figure 4, the material of this picture comes from https://www.home-for-researchers.com). It is wrapped by the astrocyte foot process and filled with CSF [13, 15]. Both animal experiments and clinical studies have shown that the polarity distribution of AQP4 is synchronized with the function of GS [21, 22]. Therefore, the destruction of PVS structure and the abnormal distribution of AQP4 polarity may be important reasons for the exchange disorder between CSF and ISF, the deposition of metabolic waste, and finally lead to or aggravate nervous system diseases.

At present, it is still a challenge to determine GS biomarkers in humans. Recently, DTI-ALPS, proposed by Taoka et al. [23], was recognized as a new noninvasive and robust method to help evaluate the function of the human GS. To date, DTI-ALPS has been employed in some studies to explore the mechanism of central nervous system-related diseases, such as Alzheimer's disease [16], Parkinson's disease [17], and type 2 diabetes mellitus [18]. The authors found that GS could reflect pathological changes, and the DTI-ALPS index was considered to be a promising biomarker of the GS in neurological disease. Many studies have revealed that the function of the GS was impaired in both haemorrhagic and ischaemic stroke [20, 24]. A large amount of metabolic waste, such as A*β* [6], tau proteins [10], lactate [25], iron [26], and proinflammatory cytokines and neurotoxic solutes [12], accumulated after stroke, resulting in brain oedema, neuroinflammation, and other pathological reactions. This accumulation and its effects are the main reasons for the high mortality and disability rate of patients with stroke [27]. The function of the GS decreased significantly after haemorrhage stroke, mainly resulting in a reduction in clearance rate of cerebral metabolic waste (such as proinflammatory cytokines and neurotoxic solutes), imbalance of central nervous system homeostasis, and so on (Figure 4, the material of this picture comes from https://www.home-for-researchers.com). Meanwhile, the abnormal structure and function of GS also accelerate the pathological development of different types of stroke [13]. Therefore, can DTI-ALPS be used to assess haemorrhagic stroke in vivo? Few studies have used DTI-ALPS to investigate patients with sICH. We assessed GS function after sICH in vivo using the DTI-ALPS index; the findings showed that the DTI-ALPS index of the lesion side was significantly decreased, while those of the contralateral cerebral hemisphere were normal. The lower DTI-APLS index indicated a weaker ability of the GS to scavenge proinflammatory cytokines and neurotoxic solutes produced after intracerebral haemorrhage. Our findings supported a previous perspective that dysfunction of the GS was involved in haemorrhagic stroke.

Interestingly, we found no significant difference in DTI-ALPS on the same side (right side) between participants in the HC and sICH groups, but it was significantly reduced on the lesion side, which indicated that GS function decreased on the ipsilateral side of the lesion but not on the contralateral side. In previous studies, the DTI parameters were only measured in the left cerebral hemisphere because the subjects were right-handed [17, 18]. Despite this, information on the DTI parameters of the right cerebral hemisphere is still lacking. As described above, blood vessels are one of the most important parts of the GS, and the PVS is a small tissue gap around the cerebral artery and vein. The bilateral cerebral hemispheres are fed by separate blood vessels. Therefore, we speculate that the separate blood supply system may be the structural basis of GS that is functionally independent, and it may explain why contralateral GS function is not involved. However, further studies that can directly observe the GS are needed to verify the GS functional properties of bilateral cerebral hemispheres.

Furthermore, the DTI-ALPS index was revealed to be significantly associated with disease duration in the current study. A previous study demonstrated that the occurrence and development of neurological disease can lead to GS dysfunction; conversely, the abnormal structure and function of the GS can also accelerate the development of the disease [20]. In other words, there may be a positive feedback relationship between GS dysfunction and disease progression. In this study, the time from intracerebral haemorrhage to DTI scanning ranged from 21 to 155 days, which may indicate that the function of GS continued to decrease during this period. This may further suggest that the function of the GS may not effectively recover in response to conventional clinical treatment. We speculated that current treatment modalities may be ineffective for a full recovery of GS function, which may be one of the potential reasons for the high recurrence rate of haemorrhage in sICH and the difficulty of effective intervention. However, we only suggest that the GS may be damaged from the perspective of imaging, but we did not verify its exact relationship from the perspective of the GS; moreover, the specific pathological mechanism is not completely clear and needs to be further studied to provide new insights that could guide the development of an effective treatment for sICH.

## 5. Limitations

Several limitations should be noted. (i) We only investigated patients with left hemisphere haemorrhagic stroke and did not recruit patients with right-sided sICH; (ii) although we obtained positive results, our sample size was still not very large; (iii) the ROI was placed manually; (iv) ICH-related clinical scores were not collected; and (v) it would be better to perform longitudinal studies to observe the changes in DTI-ALPS before and after sICH treatment.

## 6. Conclusions

The present study was designed to determine the relationship between GS function and sICH by using DTI-ALPS, and it proved that GS dysfunction is closely associated with pathophysiological changes in haemorrhagic stroke. The DTI-ALPS index can reflect the disease duration of sICH. Furthermore, this study found that GS function decreased on the ipsilateral side of the lesion but not on the contralateral side, indicating that the GS may be a separate system in the left and right cerebral hemispheres, respectively. Overall, these findings have significant implications for the understanding of sICH from a new perspective. In the future, longitudinal studies should be conducted in which a large sample of patients and patients with sICH in the left/right cerebral hemispheres are included.

## Figures and Tables

**Table 1:** Demographics and clinical data.

Variable	HCs (*N* = 31)	sICH patients (*N* = 20)	*p*
Sex (M/F)	15/16	13/7	0.24^#^
Age (years)	47.3 ± 10.9	47.8 ± 12.4	0.87^∗^
Disease duration (days)	N/A	55.6 ± 38.8	
Haemorrhage volume (mm^3^)	N/A	23.92 ± 6.67	

HCs: healthy controls; sICH: spontaneous intracerebral haemorrhage; M: male; F: female. Data are presented as range and mean ± SD. ^#^The *p* value was obtained using a chi-square test. ^∗^The *p* value was obtained by a two-sample *t*-test.

## Data Availability

The datasets generated and analysed during the current study are available from the corresponding authors on reasonable request.

## References

[1] Gross B. A., Jankowitz B. T., Friedlander R. M. (2019). Cerebral intraparenchymal hemorrhage. *JAMA*.

[2] O'Carroll C. B., Brown B. L., Freeman W. D. (2021). Intracerebral hemorrhage: a common yet disproportionately deadly stroke subtype. *Mayo Clinic Proceedings*.

[3] Weimar C., Kleine-Borgmann J. (2017). Epidemiology, prognosis and prevention of non-traumatic intracerebral hemorrhage. *Current Pharmaceutical Design*.

[4] Casolla B., Moulin S., Kyheng M. (2019). Five-year risk of major ischemic and hemorrhagic events after intracerebral hemorrhage. *Stroke*.

[5] Hostettler I. C., Seiffge D. J., Werring D. J. (2019). Intracerebral hemorrhage: an update on diagnosis and treatment. *Expert Review of Neurotherapeutics*.

[6] Iliff J. J., Wang M., Liao Y. (2012). A paravascular pathway facilitates CSF flow through the brain parenchyma and the clearance of interstitial solutes, including amyloid *β*. *Science Translational Medicine*.

[7] Iliff J. J., Lee H., Yu M. (2013). Brain-wide pathway for waste clearance captured by contrast-enhanced MRI. *Journal of Clinical Investigation*.

[8] Ahn J. H., Cho H., Kim J. H. (2019). Meningeal lymphatic vessels at the skull base drain cerebrospinal fluid. *Nature*.

[9] Louveau A., Smirnov I., Keyes T. J. (2015). Structural and functional features of central nervous system lymphatic vessels. *Nature*.

[10] Iliff J. J., Chen M. J., Plog B. A. (2014). Impairment of glymphatic pathway function promotes tau pathology after traumatic brain injury. *The Journal of Neuroscience*.

[11] Thrane V. R., Thrane A. S., Plog B. A. (2013). Paravascular microcirculation facilitates rapid lipid transport and astrocyte signaling in the brain. *Scientific Reports*.

[12] Zbesko J. C., Nguyen T. V., Yang T. (2018). Glial scars are permeable to the neurotoxic environment of chronic stroke infarcts. *Neurobiology of Disease*.

[13] Lv T., Zhao B., Hu Q., Zhang X. (2021). The glymphatic system: a novel therapeutic target for stroke treatment. *Frontiers in Aging Neuroscience*.

[14] Bu N., Khlif M. S., Lemmens R. (2021). Imaging markers of brain frailty and outcome in patients with acute ischemic stroke. *Stroke*.

[15] Opel R. A., Christy A., Boespflug E. L. (2019). Effects of traumatic brain injury on sleep and enlarged perivascular spaces. *Journal of Cerebral Blood Flow & Metabolism*.

[16] Xie L., Kang H., Xu Q. (2013). Sleep drives metabolite clearance from the adult brain. *Science*.

[17] Ma X., Li S., Li C. (2021). Diffusion tensor imaging along the perivascular space index in different stages of Parkinson’s disease. *Frontiers in Aging Neuroscience*.

[18] Yang G., Deng N., Liu Y., Gu Y., Yao X. (2020). Evaluation of glymphatic system using diffusion MR technique in T2DM cases. *Frontiers in Human Neuroscience*.

[19] Taoka T., Masutani Y., Kawai H. (2017). Evaluation of glymphatic system activity with the diffusion MR technique: diffusion tensor image analysis along the perivascular space (DTI-ALPS) in Alzheimer’s disease cases. *Japanese Journal of Radiology*.

[20] Rasmussen M. K., Mestre H., Nedergaard M. (2018). The glymphatic pathway in neurological disorders. *The Lancet Neurology*.

[21] Verkman A. S., Smith A. J., Phuan P. W., Tradtrantip L., Anderson M. O. (2017). The aquaporin-4 water channel as a potential drug target in neurological disorders. *Expert Opinion on Therapeutic Targets*.

[22] Mader S., Brimberg L. (2019). Aquaporin-4 water channel in the brain and its implication for health and disease. *Cell*.

[23] Taoka T., Ito R., Nakamichi R. (2022). Reproducibility of diffusion tensor image analysis along the perivascular space (DTI-ALPS) for evaluating interstitial fluid diffusivity and glymphatic function: CHanges in Alps index on Multiple conditiON acquIsition eXperiment (CHAMONIX) study. *Japanese Journal of Radiology*.

[24] Gaberel T., Gakuba C., Goulay R. (2014). Impaired glymphatic perfusion after strokes revealed by contrast-enhanced MRI: a new target for fibrinolysis?. *Stroke*.

[25] Lundgaard I., Lu M. L., Yang E. (2017). Glymphatic clearance controls state-dependent changes in brain lactate concentration. *Journal of Cerebral Blood Flow & Metabolism*.

[26] Tuo Q. Z., Lei P., Jackman K. A. (2017). Tau-mediated iron export prevents ferroptotic damage after ischemic stroke. *Molecular Psychiatry*.

[27] Iadecola C., Buckwalter M. S., Anrather J. (2020). Immune responses to stroke: mechanisms, modulation, and therapeutic potential. *Journal of Clinical Investigation*.

